# Protocol: Machine learning for selecting moderators in meta‐analysis: A systematic review of methods and their applications, and an evaluation using data on tutoring interventions

**DOI:** 10.1002/cl2.70009

**Published:** 2024-12-10

**Authors:** Jens Dietrichson, Rasmus Klokker, Trine Filges, Elizabeth Bengtsen, Therese D. Pigott

**Affiliations:** ^1^ Quantitative Methods, VIVE—The Danish Center for Social Science Research Copenhagen Denmark; ^2^ Administration, VIVE—The Danish Center for Social Science Research Copenhagen Denmark; ^3^ School of Public Health Georgia State University Atlanta Georgia USA

**Keywords:** machine learning, moderator analysis, tutoring, variable selection

## Abstract

Objectives

This is the protocol for a Campbell systematic review. The objectives are as follows: The first objective is to find and describe machine and statistical learning (ML) methods designed for moderator meta‐analysis. The second objective is to find and describe applications of such ML methods in moderator meta‐analyses of health, medical, and social science interventions. These two parts of the meta‐review will primarily involve a systematic review and will be conducted according to guidelines specified by the Campbell Collaboration (MECCIR guidelines). The outcomes will be a list of ML methods that are designed for moderator meta‐analysis (first objective), and a description of how (some of) these methods have been applied in the health, medical, and social sciences (second objective). The third objective is to examine how the ML methods identified in the meta‐review can help researchers formulate new hypotheses or select among existing ones, and compare the identified methods to one another and to regular meta‐regression methods for moderator analysis. To compare the performance of different moderator meta‐analysis methods, we will apply the methods to data on tutoring interventions from two systematic reviews of interventions to improve academic achievement for students with or at risk‐of academic difficulties, and to an independent test sample of tutoring studies published after the search period in the two reviews.

## 1 BACKGROUND

Moderator analyses aim to examine heterogeneity in meta‐analyses. To reduce the risk of inadvertently reaching spurious or researcher‐favoured conclusions, methodological guidelines typically recommend pre‐specifying a limited number of moderators to include in a meta‐regression (e.g., Deeks et al., 2021; Higgins et al., 2019; Pigott & Polanin, 2020). However, the purpose of the moderator analysis may be to generate new hypotheses to test in new trials, or to find good predictions of the effect sizes of interventions to aid statistical power analyses. That is, the purpose of the analysis may be exploratory rather than confirmatory.

During recent decades, the use of machine and statistical learning (ML) methods have increased dramatically in the health, medical, and social sciences, with applications ranging from prediction to exploration of effect heterogeneity (e.g., Athey, 2017; Grimmer et al., 2022; Mullainathan & Spiess, 2017; Verhagen, 2022; Waring et al., 2020). Many ML methods are designed to handle situations when the ratio of interesting predictors to the number of observations is high—which is often the case in meta‐analysis—and are able to incorporate flexible functional forms (Dusseldorp et al., 2014; van Lissa, 2017), while meta‐regression models used in moderator analysis are often restricted to linear regressions (Higgins et al., 2019). ML methods involve measures to reduce the risk of overfitting (Dusseldorp et al., 2014; van Lissa, 2017)—that is, the risk of training a model to fit noise in the data instead of extracting the model structure and thereby causing poor predictions outside the training data set—or induce benign overfitting (Bartlett et al., 2021). Thus, ML methods may be suitable for exploratory moderator analyses, and complement other methods for examining heterogeneity and generating new hypotheses (e.g., meta‐regressions, mixed methods synthesis, and coproduction activities with stakeholders).

The applications of ML methods to moderator meta‐analysis are scattered across fields (see e.g., Bonapersona et al., 2019; Curry et al., 2018; Dusseldorp et al., 2014; Roberts et al., 2019; Sheeran et al., 2019; and Williams et al., 2022 for applications), which may imply that there is a potential for cross‐field learning. Moreover, the number of studies comparing different ML methods in the context of meta‐analysis seem to be very few (van Lissa, 2017). To the best of our knowledge, no study have compared ML methods for moderator meta‐analysis using non‐simulated review data.

In this meta‐review, we will describe and compare ML methods developed for moderator meta‐analysis, and how meta‐analyses of health, medical, and social science interventions have applied these methods. There is, to our knowledge, no agreed upon definition of what constitutes ML methods and how these methods differentiate themselves from more traditional statistical modelling techniques, such as linear regression models. For example, James et al. (2017) described statistical learning as ‘a set of tools for modelling and understanding complex data sets’ (p. vii). This definition includes methods like linear and logistic regressions, which are common in moderator meta‐analysis. Other authors have made a distinction between methods that assume a statistical model and methods that try to find a model that fits the data well, for example, by using a data‐driven selection of which variables to include in the model (e.g., Breiman, 2001a; Bzdok et al., 2018).

We will follow this second distinction and include all statistical methods that perform data‐driven variable selection in some form. That includes for example methods using penalized regressions—such as the Least Absolute Shrinkage and Selection Operator (LASSO; Tibshirani 1996)—and tree based methods, e.g., classification trees and random forests. While polynomial regressions and spline regressions are examples of statistical learning methods (James et al., 2017) and allow for more flexible functional forms than regular linear meta‐regressions, they do not select among moderators, and we therefore exclude meta‐analytic studies using them. We will furthermore not include information‐theoretic approaches for selecting moderators in regular linear meta‐regressions (e.g., Cinar 2021). That is, the variable selection should be performed by the method, not the reseracher after comparing results to some outside criteria. If the included studies examine performance differences between ML methods and linear regression methods, we will describe the results of these examinations in our review.

We will also investigate how ML methods can help researchers formulate new hypotheses or select among existing ones using a data set of tutoring interventions from two previous systematic reviews (Dietrichson et al., 2020; Dietrichson et al., 2021). The reviews found both large average effect sizes on standardized tests in mathematics and reading, as well as substantial unexplained heterogeneity of the effect sizes in a category of interventions almost exclusively made up by tutoring interventions. Including moderators of the group size, study design, subject, test type, mean grade, and duration of the intervention reduced the heterogeneity very little. The only statistically significant moderator was the mean grade. Thus, tutoring has the potential to make substantial improvements of academic achievement for at‐risk students, but it is unclear why the effect sizes vary so much. We hope that by using ML methods to examine this heterogeneity, we may generate new hypotheses of why tutoring effect sizes differ, and of how more effective tutoring interventions can be designed.

### 1.1 Why it is important to do this review

Examining the heterogeneity of effect sizes has been a centrepiece of meta‐analysis since its inception. Over time the emphasis has moved from estimating the extent of heterogeneity to also encompass explaining heterogeneity, using moderator variables such as study design, intervention characteristics, etc. (Pigott & Polanin, 2020; Tipton et al., 2019). To investigate and explain heterogeneity, researchers have typically applied meta‐regression models or subgroup analyses. However, such methods suffer from many of the same drawbacks as regression‐based models do outside the context of meta‐analysis. Furthermore, the small sample sizes often found in meta‐analytic datasets severely limit the amount of moderators that can be included in the meta‐regression model, and may be a hindrance to conducting exploratory moderator analysis or result in model specifications that are overly parsimonious.

Current practices typically rely on linear models which may impose unreasonable assumptions of linearity on associations between moderators and effect sizes. While nonlinear associations can be accounted for, to some extent, for example, via inclusion of polynomial terms, such terms must still be pre‐specified. They must be included alongside the non‐transformed moderator, thus using precious degrees of freedom. Also when including polynomial terms, the meta‐regression models are still restricted to being linear in parameters.

In contrast, several modelling techniques have been developed in the ML literature that address these limitations. Methods such as LASSO regression, decision trees, random forests and neural nets have attracted much attention due to their ability to provide more accurate predictions. It is of special interest that these modelling techniques often provide superior predictive performance in settings that are known to hamper traditional statistical modelling techniques, such as very high dimensional settings (e.g., GWAS; Li et al., 2014), in which the number of predictors is as high or higher than the number of observations, and data with highly nonlinear associations. Additionally, many ML methods do not require that the researcher specifies nonlinear associations or interactions between variables, as those are, to some extent, detected by the models. Thus, ML methods have the potential to overcome several of the limitations imposed by traditional meta‐regression models.

One common drawback of ML methods is that they are difficult to interpret (Athey, 2017; Verhagen, 2022). For example, most ML methods do not provide ways to assess exactly how a moderator is associated with effect sizes, as beta coefficients in a regression model do. However, it is possible to extract information regarding how ‘important’ the variable is, according to the model, when attempting to predict the outcome (by e.g., variable importance rankings and partial dependence plots; van Lissa, 2020a; Williams et al., 2022).

As of yet, there are, however, few comparisons and reviews about how ML methods can be used in exploratory moderator analyses. We are only aware of one earlier comparison of ML methods for meta‐analysis: van Lissa (2017) compared metaCART and MetaForest using simulated data. MetaCART was first developed by Dusseldorp et al. (2014) and uses decision trees. The method builds on the Classification and Regression Tree (CART) algorithm first developed by Breiman (1984), which is adapted to meta‐analysis. MetaForest, developed by van Lissa (2017), instead builds on a random forest algorithm (Ho et al., 1995; Breiman, 2001b) adapted to meta‐analysis.

Our meta‐review will provide an overview of available ML methods for moderator analyses and how these methods have been applied. We will also investigate how ML methods perform in the context of exploratory moderator meta‐analysis using non‐simulated data. A major advantage of using simulated data is that the correct model is known, and that methods can be compared by how well they can approximate the correct model. However, we are interested in examining how ML methods can help researchers formulate new hypotheses or select among existing ones. We are less interested in answering which method is the best, which is likely to be context‐specific. For this exploratory purpose, we believe non‐simulated, real‐world data will be more informative.

Our review will contribute to the understanding of how tutoring interventions work, and in which forms and contexts they are most effective. It has long been known that tutoring can be highly effective (e.g., Bloom 1984; Cohen et al., 1982; Ritter et al., 2006; Nickow et al., 2024). Reviews comparing the effects of interventions for at‐risk students often find the largest effect sizes for intensive, small‐group instruction by adults (e.g., Baye et al., 2019; Dietrichson et al., 2017; Dietrichson et al., 2020; Dietrichson et al., 2021; Fryer, 2017; Neitzel et al., 2022; Slavin et al., 2011). The bulk of these interventions would fit our definition of tutoring. The magnitude of the effects in the most effective tutoring interventions indicate that they would be able to eradicate a large proportion of the typical achievement gaps between, for example, high and low socioeconomic status (SES) students (e.g., Blachman et al., 2004; Bøg et al., 2021), at least in the short‐term and if given only to low SES students.

However, reviews typically also find substantial heterogeneity of the effect sizes. In the few reviews that have examined the heterogeneity using meta‐regressions, few moderators showed strong associations with effect sizes and the unexplained heterogeneity remained substantial (Dietrichson et al., 2017; Dietrichson et al., 2021; Nickow et al., 2024; Ritter et al., 2009; Wanzek et al., 2016). Many moderators that theoretically could explain the heterogeneity have never been examined in a moderator analysis. In sum, tutoring has the potential to make substantial improvements of academic achievement for at‐risk students but more knowledge about the heterogeneity of effect sizes is needed. ML methods may help us generate new hypotheses of why tutoring effect sizes differ, and in turn, how more effective tutoring interventions can be designed.

### 1.2 Description of included tutoring interventions

To examine how ML methods perform using real intervention data, we intend to apply the methods to data on tutoring interventions. This section briefly describes how we define tutoring and potential reasons why tutoring effect sizes are heterogeneous.

Tutoring is an instructional method in which students receive instruction either one‐to‐one, or in small groups, by for example teachers, special educators, paraprofessionals, researchers, volunteers, or peers (e.g., Cohen et al., 1982; Juel, 1996). Tutoring is sometimes also called small‐group or one‐to‐one tuition (e.g., Education Endowment Foundation, 2022). Tutoring can in principle be used in any subject, and with any type of student, but it is often used for remedial purposes for students with, or at‐risk, of academic difficulties.

We will use a data set of tutoring interventions collected by Dietrichson et al. (2020, 2021). These two reviews had identical inclusion criteria except that Dietrichson et al. (2020) was limited to students in Grades 7 to 12 and Dietrichson et al. (2021) was limited to students in kindergarten to Grade 6. The search and screening processes were conducted jointly for the two reviews. We describe the data set in more detail in the Types of studies section below.

In line with these two reviews, we will define tutoring as instruction in groups of no more than five students by adults. That is, we will not include peer‐tutoring in our definition of tutoring. Adults are defined by being older than secondary school students (i.e., they are not in Grade K‐12). Furthermore, we will include interventions aiming to improve math or reading/literacy skills (or both), and exclude mentoring interventions in which the focus was not math or reading, but for instance social‐emotional skills. The mentoring interventions were relatively few, and we believe it will be advantageous to focus on a more homogeneous set of interventions. For the same reason, we will restrict the analysis to interventions that changed only one instructional method (i.e., there were no co‐interventions), and use only end‐of‐intervention tests.

#### 1.2.1 Potential reasons for the heterogeneity of tutoring effect sizes

Tutoring includes several features that may be beneficial for student learning. The small‐group instruction allows the tutor to individualize the pace and the support, which should make it easier to make instruction challenging but not too difficult (i.e., to keep the student in the ‘Zone of Proximal Development’ as advocated by Vygotsky, 1978). In a similar vein, the format facilitates frequent feedback from the tutor to the tutee and vice versa, which is emphasized by pedagogical theories as important for learning (e.g., Hattie & Timperley, 2007). Furthermore, the instructional mode includes social interaction with a, hopefully, supportive role model. Interacting with the tutor may imply that the students get attention and encouragement, which may influence both motivation and self‐efficacy (Juel, 1996; Neitzel et al., 2022). Tutoring sessions furthermore provide opportunities to practice the regulation of behaviour, which social learning theories emphasize as important for learning (e.g., Bandura, 1986). Tutoring may also train more domain‐general cognitive skills such as attention, and working and short‐term memory (Goldstein, 1976; Share, 1995). These cognitive skills are believed to be important for all kinds of academic learning (e.g., Diamond, 2013).

Below, we present potential explanations of heterogeneity related to the form and content of tutoring programs, the target group characteristics, the control group instruction, and the study characteristics. These moderators may, in turn, interact with one another.

*Program form*. By our definition, tutoring programs all share the small‐group format, but group sizes, the type of tutors, and dosage (duration, frequency, intensity) may differ.

*Program content*. Tutoring programs may be more or less manual‐based (or structured), target different areas of math and reading, and employ specific methods or techniques (e.g., multisensory methods).

*Target group characteristics*. The age of the students receiving the tutoring, and the severity of the difficulties they face may matter for effect sizes.

*Control group instruction*. The included studies in this review all compared a tutoring program to some form of treatment‐as‐usual condition for the control group. The quality and content of this treatment‐as‐usual condition may, however, differ quite a lot between studies, which may, in turn, explain why effect sizes are heterogeneous.

*Study characteristics*. The heterogeneity may also be a consequence of how the intervention was implemented, and how the study was conducted (e.g., a randomized controlled trial or a quasi‐experimental study, recruitment practices, and the number of involved schools).

The above list indicates many theoretical reasons to expect heterogeneity of tutoring effect sizes. In turn, effect sizes may depend on a large number of moderators, and on both complex interactions among these moderators and nonlinear effects of, for example, dosage. Incorporating variables representing all these possible reasons in regular meta‐regressions would imply including a very large set of explanatory variables, which may be infeasible even with our relatively large set of interventions (91 studies and 551 effect sizes). For these reasons, we believe that ML methods may be useful for analyzing the heterogeneity of tutoring effect sizes, and that our data set of tutoring interventions will provide a good testing ground for ML methods in moderator meta‐analyses.

## 2 OBJECTIVES

The objective of this meta‐review is threefold. The first objective is to find and describe machine and statistical learning (ML) methods designed for moderator meta‐analysis. The second objective is to find and describe applications of such ML methods in moderator meta‐analyses of health, medical, and social science interventions. These two parts of the meta‐review will primarily involve a systematic review and will be conducted according to guidelines specified by the Campbell Collaboration (MECCIR guidelines). The outcomes will be a list of ML methods that are designed for moderator meta‐analysis (first objective), and a description of how (some of) these methods have been applied in the health, medical, and social sciences (second objective).

The third objective is to examine how the ML methods identified in the meta‐review can help researchers formulate new hypotheses or select among existing ones, and compare the identified methods to one another and to regular meta‐regression methods for moderator analysis. We are interested in examining how ML methods can supplement more traditional ways of conducting exploratory moderator analysis. To compare the performance of different moderator meta‐analysis methods, we will apply the methods to data on tutoring interventions from two systematic reviews of interventions to improve academic achievement for students with or at risk‐of‐academic difficulties (Dietrichson et al., 2020, 2021), and to an independent test sample of tutoring studies published after the search period in the two reviews. We will compare the models over three dimensions:

- Variable selection: are there substantive differences between the variables selected by the ML methods, and the variables included on theoretical grounds in the meta‐regressions?
- Heterogeneity explanatory performance: how much heterogeneity is left unexplained by the methods?
- Predictive performance: how well do the methods predict effect sizes in‐sample (i.e., in the data set from Dietrichson et al., 2020, 2021) and out‐of‐sample (i.e., in the independent test sample)?

We want to emphasize that the purpose of this comparison is not to select the best method. The best method is likely to be context‐specific, and our data and purpose represent just one example of a context. Instead, the comparison is intended to help researchers get an overview of ML methods and how they can be implemented, and help us discuss the overarching research question for this part of the review: how can ML methods help researchers generate new hypotheses about why effect sizes are heterogeneous, and help select the most important hypotheses among existing ones?

## 3 METHODS

### 3.1 Criteria for considering studies for this meta‐review

#### 3.1.1 Types of studies

In this meta‐review, we will be including meta‐analyses within the health, medical, and social sciences that apply ML methods in moderator analysis. Included meta‐analyses should apply the ML methods to effect sizes from studies of intervention effects. We will exclude meta‐analyses of correlational studies and those that use simulated data to examine ML methods.

We will distinguish ML methods from other methods for prediction and exploration by requiring that the included methods perform data‐driven variable selection in some form. That includes, for example, methods using regularization (or penalized regressions)—such as LASSO—and tree‐based methods, for example, classification trees and random forests.

This criterion excludes methods like polynomial regressions and spline regressions. These methods allow for more flexible functional forms than regular linear meta‐regressions but do not perform data‐driven selection of moderators. We will furthermore not include information‐theoretic approaches for selecting moderators in regular linear meta‐regressions (e.g., Cinar, 2021). That is, the variable selection should be performed by the method, not the reseracher after comparing results to some outside criteria.

We will also include studies that describe the development of ML methods intended for moderator meta‐analysis, regardless of the field.

We will place no additional restrictions on the type of participants, interventions, outcome measures, duration of follow‐ups, or types of settings, besides those given by the restriction to health, medical, and social science interventions.

#### 3.1.2 Data set of tutoring interventions

We will use a merged data set of tutoring interventions collected by two previously published Campbell reviews: Dietrichson et al. (2020, 2021). In brief, the inclusion criteria for these reviews were:

- Population: The population eligible for the review included students attending regular schools in kindergarten to Grade 12 in an OECD country, who were having academic difficulties, or were at risk of such difficulties.
- Intervention: Eligible interventions sought to improve academic skills, were conducted in schools during the regular school year, and were targeted (selected or indicated).
- Comparison: Included studies used an intervention‐control group design or a comparison group design: randomized controlled trials (RCT); quasi‐randomized controlled trials (QRCT); and quasi‐experimental studies (QES).
- Outcomes: Included studies used standardized tests in reading or mathematics. These tests included norm‐referenced tests (e.g., Gates‐MacGinitie Reading Tests and Star Math), state‐wide tests (e.g., Iowa Test of Basic Skills), and national tests (e.g., National Assessment of Educational Progress, NAEP). Tests of specific domains (e.g., vocabulary, fractions) as well as more general tests, which tested several domains of reading or mathematics, were both included.

These criteria included many more types of interventions than tutoring interventions and we will use only a subset of the studies included in reviews (for more details about the inclusion criteria, please see Dietrichson et al., 2020, 2021).

As mentioned, we will define tutoring as instruction in groups of no more than five students by adults (persons older than secondary school students), and only include interventions aiming to improve math or reading/literacy skills (or both). Dietrichson et al. (2020, 2021) examined a broader small‐group instruction category that also included ‘mentoring’ interventions in which the focus of instruction was not necessarily math or reading, but for instance social‐emotional skills. To further make the data set more homogeneous, we will restrict the analysis to interventions that changed only one instructional method. That is, interventions in which the treatment group received tutoring and no co‐intervention, and the control group did not receive any intervention but some form of treatment as usual. Moreover, we will include only end‐of‐intervention tests, that is, tests made within 3 months after the intervention ended. Longer follow‐ups will be excluded to not introduce confounding by later interventions by, for example, schools and parents. Lastly, we will only include effect sizes that had a sufficiently low risk of bias (i.e., were not rated critical risk of bias). For the ratings of the included studies and effect sizes in these reviews, see Dietrichson et al. (2020, 2021).

The tutoring effect sizes in Dietrichson et al. (2020, 2021) were calculated by comparing a treatment to a control (treatment as usual) condition on a standardized test score in reading or mathematics. Almost all studies used continuous outcome measures, and we will therefore use standardized mean differences (SMDs) as the outcome variable, estimated as Hedges’ *g* (Lipsey & Wilson, 2001). The few studies that did not report continuous outcomes will be transformed into SMDs using the Cox transformation‐method recommended by Sanchez‐Meca et al. (2003) and be analysed together with the continuous outcomes. We will follow Dietrichson et al. (2021) in coding all effect sizes so that positive effect sizes imply beneficial effects of the intervention, and in how we adjust effect sizes from cluster‐assigned treatments.

With these inclusion criteria, the main data set will contain 91 studies and 551 effect sizes. As mentioned, we will also use an independent test data set in the analyses. We will attempt to find 10 new tutoring studies to be included in this data set. We describe how we will find these studies in the subsection Searching for an independent test sample below.

### 3.2 Search methods for identification of studies

The main search strategy has a two‐pronged objective. First, to find all ML methods developed for moderator meta‐analysis. Second, to find all meta‐analyses that have used a ML method to examine effect size heterogeneity in health, medicine, and social science interventions.

#### 3.2.1 Electronic searches

We plan to conduct searches in the following databases (search platform in parentheses):

- Academic Search Premier (EBSCO‐host).
- ERIC (EBSCO‐host).
- PsycInfo (EBSCO‐host).
- Socindex (EBSCO‐host).
- Teacher Reference Center (EBSCO‐host).
- EconLit (EBSCO‐host).
- Science Citation Index (Web of Science).
- Social Science Citation Index (Web of Science).
- Sociological Abstracts (ProQuest).
- PubMed.

##### Search terms

Below we show an example of our search strategy with two facets. The first facet intends to capture meta‐analyses and the second ML methods. As known ML methods for moderator meta‐analysis have names consisting of the prefix ‘meta’ and the name of a ML method as suffix (e.g., metaCART, MetaForest), we will use left truncation in databases where it is possible, and further specify the search terms in databases, like PubMed, which do not allow left truncation (e.g., *CART and metaCART in the example below). The strategy includes proximity searching, which not all databases allow (e.g., PubMed, Web of Science). We will omit this part when searching databases in which proximity search is not possible. A full description of modifications used in the searches of the specific databases will be added to the final review. An example of our search strategy from ERIC is shown below.

**Table:** 

S1	TI AB ‘meta analys*’ OR meta‐analys* OR metaanalys* OR meta‐analyt* OR ‘meta analyt*’ OR metaanalyt* OR (meta* N5 (moderator OR subgroup OR ‘sub‐group’)) OR (review N5 (moderator OR subgroup OR ‘sub‐group’)) OR (meta* N5 heterogeneity) OR (review N5 heterogeneity)
S2	DE Meta Analysis
S3	S1 OR S2
S4	TI AB ‘machine learning*’ OR machine‐learning* OR ‘statistical learning*’ OR statistical‐learning* OR ‘supervised learning*’ OR ‘semi‐supervised learning*’ OR ‘self‐supervised learning*’ OR ‘unsupervised learning*’ OR ‘artificial intelligence*’ OR ‘variable select*’ OR ‘high dimensional*’ OR high‐dimensional* OR ‘dimension* reduction*’ OR ‘deep learning*’ OR ‘deep model’ OR ‘neural net*’ OR LASSO* OR ‘shrinkage*’ OR ‘random forest*’ OR random‐forest* OR ‘decision tree*’ OR ‘classification tree*’ OR ‘binary classification’ OR ‘multi‐class classification’ OR ‘multinomial classification’ OR ‘support vector machine*’ OR regularization* OR regularized* OR ‘penalized regression*’ OR ‘elastic net*’ OR ‘boosted model*’ OR ‘gradient boost*’ OR boosting OR ‘gradient descent’ OR ‘prototype model*’ OR K‐nearest* OR k‐nearest* OR ‘stacked ensemble*’ OR ‘ensemble method*’ OR K‐mean* OR kmean* OR K‐median* OR k‐median* OR ‘XGBoost*’ OR ‘LightGBM*’ OR *CART OR ‘metaCART’ OR meta‐forest* OR ‘meta forest*’ OR metaforest* OR kernel* OR ‘tree based’ OR tree‐based OR ‘regression tree*’ OR ‘regression‐tree*’ OR AdaGrad OR ‘agglomerative clustering’ OR ‘divisive clustering’ OR ‘hierarchical clustering’ OR ‘centroid‐based clustering’
S5	DE Artificial Intelligence
S6	S4 OR S5
S7	S3 AND S6

#### 3.2.2 Searching other resources

##### Citation tracking

We will conduct both backward and forward citation tracking of the included studies. As we expect that the method used in the moderator analysis is not always mentioned in the title or abstract of a study, our electronic database search may miss relevant studies. However, studies should reference the ML methods they use, and at least some mention the methods in the abstract. We should therefore be able to find studies developing the ML method through backward citation tracking. Then we can use forward citation tracking of the studies developing the method to locate meta‐analyses using the method in question that do not mention the method in the title or abstract. Backward and forward citation tracking will therefore be extra important in this review.

##### Contacting international experts

We will contact international experts to identify additional studies and provide them with the inclusion criteria for the review along with the list of included studies, asking for any other published, unpublished or ongoing studies relevant to the review. We will primarily contact corresponding authors who have used ML methods in their moderator meta‐analysis.

##### Search for systematic reviews

We will search for other relevant systematic reviews in the following resources:

- Campbell Systematic Reviews—https://onlinelibrary.wiley.com/journal/18911803.
- Cochrane Library—https://www.cochranelibrary.com/.
- Centre for Reviews and Dissemination Databases—https://www.crd.york.ac.uk/CRDWeb/.
- EPPI‐Centre Systematic Reviews—Database of Education Research https://eppi.ioe.ac.uk/cms/Databases/tabid/185/Default.aspx.

##### Hand search

We will conduct a hand search of the following journals, which may publish methods articles, to increase the probability that we find all relevant and recent studies:

- *Research Synthesis Methods.*
- *Psychological Methods.*
- *Statistics in Medicine.*
- *Epidemiologic Methods.*
- *Journal of Educational and Behavioral Statistics.*
- *Journal of Economic Surveys.*
- *Sociological Methods & Research.*

Based on the results of our screening process, we will also hand search the five journals that are most frequently represented among our included studies. Thus, we will hand search in total 12 journals.

##### Grey literature search

We will search specifically after three types of grey literature: working papers, reports, and dissertations. Some of the bibliographic databases also cover grey literature (e.g., ERIC). We will search the following resources for grey literature:

- ProQuest Dissertations & Theses Global (dissertations) (EBSCO‐host).
- EBSCO Open Dissertations (dissertations) (EBSCO‐host).
- Open Grey (reports, working papers, dissertations)—http://www.opengrey.eu/.
- Google Scholar (reports, working papers, dissertations)—https://scholar.google.com/.
- Google searches (reports, working papers, dissertations)—https://www.google.com/.
- Social Care Online (reports, working papers, dissertations, systematic reviews)—https://www.sciesocialcareonline.org.uk/.
- Social Science Research Network (working papers)—https://www.ssrn.com/index.cfm/en/.
- PsyArxiv—https://psyarxiv.com/.
- SocArxiv—https://osf.io/preprints/socarxiv.
- MedArxiv—https://www.medrxiv.org.
- EdArxiv—https://edarxiv.org/.
- RePEc (Research Papers in Economics)—https://ideas.repec.org/search.html IEEE Xplore—https://ieeexplore.ieee.org/Xplore/home.jsp.

Further resources for identifying grey literature may be added during the search process. A final list of grey literature resources will be included in the appendix of the review.

#### 3.2.3 Searching for an independent test sample

In one analysis, we will use a sample of tutoring studies that were not included in Dietrichson et al. (2020, 2021) to examine the predictive performance of the included ML‐methods. To find this sample, we will use the search strategy in Dietrichson et al. (2020, 2021) and augment the strategy with the search terms ‘tutoring,’ ‘small group,’ and ‘small‐group’ (i.e., title or abstract must include one of these terms). We will search a period starting in 2019 (i.e., after the search was conducted in Dietrichson et al., 2020, 2021) and restrict the databases to ERIC, which we deem to be the most central database for tutoring interventions.

When screening these studies, we may use the priority screening function in EPPI to reduce the resources used to find a small sample of studies. We will stop when we have found 10 studies that match our inclusion criteria (as described in section *Data set of tutoring interventions*), has sufficiently low risk of bias, and contains enough information to be meta‐analysed (note that a study may be reported in more than one record).

### 3.3 Data collection and analysis

#### 3.3.1 Selection of studies

We will divide the screening process for the ML methods and moderator meta‐analyses into two stages: (1) screening on title and abstract, and (2) screening on full text. We will make use of independent double screening at both stages to ensure the quality of the screening process and reduce potential errors (Polanin et al., 2019; Stoll et al., 2019). The screeners (primarily research assistants) will be blind to each other's work until comparing final judgements. If the two screeners cannot agree on the inclusion/exclusion of a specific reference, the reference will be sent to one of the review authors for final judgement.

We will conduct a pilot‐screening for each screening stage and each screener. In the pilot screening of title and abstract, the review team will screen and compare 80–100 references. The review team will then discuss and resolve potential disagreements and uncertainties. If the interrater agreement is above 90% in the pilot, the screeners will continue to screen the rest of the references. If the interrater agreement is below 90% in the first pilot, the review team members will perform a second pilot screening to ensure reliability. At the full text stage of the screening process, the pilot will consist of 8‐10 studies. The pilot procedure at second level is otherwise identical to the process described for first level. The review team will meet with regular intervals to discuss uncertainties and minimize ‘coders drift’ (Polanin et al., 2019).

We will present the overall search and screening process in a flow chart in the final review. During the screening process, none of the review authors or review team members will be blind to the authors, journals, or institutions responsible for the publication of eligible studies.

#### 3.3.2 Data extraction and management

Two members of the review team will independently extract and code data from the included studies of ML methods, and the included moderator meta‐analyses of health, medical, and social science interventions. Before that, the coding tool will be piloted and potentially revised. From all included studies, we extract data on publication characteristics, study characteristics, and characteristics of the ML method. If any disagreement or uncertainty emerges during the data extraction process, a third reviewer with the appropriate expertise will be consulted.

The tutoring studies have been coded and extracted by at least two members of the review team in Dietrichson et al. (2020, 2021). We will however re‐visit these studies and code more tutoring‐specific moderators. Any extra coding will be done in duplicate by at least two review team members. All extracted data will be stored electronically using EPPI Reviewer 4 and Microsoft Excel.

#### 3.3.3 Data synthesis

Our first objective is to find and describe ML methods designed for moderator meta‐analysis. We will list and describe the ML methods that have been used for moderator analysis, focusing on how they can be used to perform variable selection.

The second objective is to find and describe applications of ML methods in moderator meta‐analyses of health, medical, and social science interventions. We will provide descriptive statistics about the health, medical, and social science reviews using these methods (e.g., area, type of interventions, outcome measures, number of included studies and effect sizes), the ML methods they used (method name, type of ML method), and the results (heterogeneity parameters, model performance measures). We will also describe how the ML methods were used in the moderator analysis (e.g., did they use variable importance rankings or partial dependence plots to perform variable selection?) and whether ML methods were compared to regular meta‐regressions methods. Besides descriptive statistics (e.g., heterogeneity statistics from ML method and meta‐regression, if available), we will discuss how ML methods have been applied, and provide assessements from the review authors on how ML methods fared in comparison to regular meta‐regressions (if such assessments are available).

Our third objective is to compare the performance of ML methods identified in the meta‐review to one another, and to regular meta‐regression methods for moderator analysis. As mentioned in the Objectives‐section, we will use a data set of tutoring interventions to conduct three types of comparisons:

- Variable selection: are there substantive differences between the variables selected by the ML methods, and the variables included on theoretical grounds in the meta‐regressions?
- Heterogeneity explanatory performance: how much heterogeneity is left unexplained by the methods?
- Predictive performance: how well do the methods predict effect sizes in‐sample and out‐of‐sample?

The analysis of effect sizes from tutoring interventions and the comparison of ML methods cannot be fully specified before we know what ML methods we will compare. However, based on the methods we are aware of (e.g., metaCART and MetaForest), we anticipate a number of issues. First, we discuss how to choose a benchmark meta‐regression specification. We then discuss variable selection and how to assess and interpret differences between the variables selected by the ML methods, and the variables included on theoretical grounds in meta‐regressions. This issue is related to how we use the results from the ML models, and also to the measures we use to asses heterogeneity, which is discussed in the same subsection. Related to the third comparison, we also discuss predictive performance, in‐sample and out‐of‐sample. The last two subsections contain information on how we will implement the ML methods and how we will deal with missing data and interdependent effect sizes.

##### Benchmark meta‐regression specifications

First of all, we need a specification representing ‘traditional’ meta‐regression approaches to moderator analysis. We take as our starting point the specification reported in column 2 of table 7 in Dietrichson et al. (2021), which was the most extensive specification used to examine small‐ and medium‐group instruction in that review. The specification included the following moderators: indicators for group sizes of one, two or three, and four or five students (with medium‐group size interventions as the reference category, which will not be included in the current review), an indicator for QES designs (with RCTs as the reference category), an indicator for math tests (with reading tests as the reference category), general tests (with tests of subareas of reading and math as the reference category), the mean grade‐level of the participants, and the duration of the intervention. Using one group size indicator as the reference category, there were seven moderators plus an intercept, which is in between the average number of moderators included in meta‐regressions in the Psychological Bulletin (mean = 5.5) and Review of Educational Research (mean = 9.7), as reported by Tipton et al. (2019). The benchmark specification will, in this sense, be fairly typical of meta‐regressions in journals relevant to education interventions.

The benchmark specification is thus pre‐specified and an example of a confirmatory type of specification. However, the review concerns exploratory moderator analyses, and the benchmark specification may undervalue the potential of meta‐regressions to explain heterogeneity. With 91 studies and 7 moderators, the specification could include two more moderators and still follow the rule of thumb of at least ten studies per moderator given by Cochrane Handbook for Systematic Reviews of Interventions (Deeks et al., 2021). Furthermore, the moderators included in the benchmark specification all have a theoretical motivation but they were also chosen because they had no missing observations in the data set used in Dietrichson et al. (2021). As discussed further below, we will impute missing values in this review. As the coding in Dietrichson et al. (2020, 2021) was not solely concerned with tutoring interventions, we will also re‐code the included studies regarding information that we believe may be important for effect size heterogeneity in tutoring interventions. For these reasons, we will also report results from an augmented meta‐regression specification that includes the moderators we believe have the highest chance of explaining the heterogeneity of effect sizes. We will run this specification first, before we implement any ML methods.

We will use the correlated‐hierarchical effects (CHE) model developed by Pustejovsky and Tipton (2022) to implement our meta‐regressions. As dependencies between effect sizes that arise because the same students are tested with different tests (‘correlated effects’) and because different samples are included in the same study (‘hierarchical effects’) are present in our sample, the ability to take these sources of dependencies into account is a major advantage of the CHE model. The CHE model also combines multi‐level modelling with robust variance estimation of the standard errors, which will allow us to examine both within‐ and between‐study heterogeneity while reducing the risk of type I errors (i.e., falsely rejecting the null hypothesis of no association between effect sizes and moderators).

We will use the *clubSandwich* (Pustejovsky, 2022) and *metafor* (Viechtbauer, 2010) packages in R to estimate the CHE models. We will use the *clubSandwich* package to specify the correlation structure between effect size estimates within studies. Then, we will estimate the random effects variance components, inverse‐variance weight matrices, and the meta‐regression coefficients using the restricted maximum likelihood (REML) procedure in the *metafor* package. We will calculate confidence intervals for the coefficients based on the RVE standard errors obtained from the *clubSandwich* package (the RVE procedure only concerns the standard errors, and not the estimation of the variance components). These standard errors are adjusted for small‐sample bias as suggested by Tipton (2015) and Tipton and Pustejovsky (2015). We intend to report 95% confidence intervals for all metaregression analyses. The CHE model requires a pre‐specified value for the correlation between pairs of effect sizes from the same study (*ρ*). We will choose 0.6, as suggested by Pustejovsky and Tipton (2022), and test whether our results are sensitive to choosing different values of ρ, by also fitting CHE models with *ρ* set to 0.4 and 0.8.

##### Moderator selection and heterogeneity measures

ML methods may select the same moderators as those included in the benchmark and augmented specifications. We will interpret this outcome as evidence that ML methods, in the case of tutoring interventions, were not helpful in finding additional important moderators or selecting among moderators. That is, the methods did not help us generate new hypotheses about the reasons for effect size heterogeneity, and did not help us select the most important hypotheses among existing ones. Such a result would not necessarily reflect poorly on the ML methods. If the ML methods identify the same moderators as a researcher‐selected specification that explains the heterogeneity well, then finding the same moderators would be an impressive performance. Furthermore, ML methods may still be useful in such cases, in the sense that they provide a data‐driven validation of the theory‐driven selection of moderators.

If the ML methods instead select different moderators than the ones included in the benchmark or augmented specification, then this may be a sign that ML methods can improve exploratory moderator analyses. However, finding examples of possibly important moderators that were not selected by us will not constitute conclusive evidence of the superiority of ML methods or that a new hypothesis about effect size heterogeneity should be formulated. It may, for example, be the case that the ML methods select moderators that are interchangeable to the researcher‐selected moderators in the sense that they capture the same underlying variation. Examining the correlations between selected and not selected moderators may, therefore, yield additional information.

We also need metrics to compare the performance of the methods with one another, and to examine whether there is substantial unexplained heterogeneity. Commonly reported heterogeneity parameters in meta‐regressions are the *Q*‐statistic, *τ* ^2^ and *τ* (the between‐study variance and standard deviation), and the *I* ^2^. The CHE model may also be used to decompose the total variance in a within‐ (denoted *ω* ^2^) and a between‐study component (*τ* ^2^), and to calculate the total standard deviation as *σ* = √(*ω* ^2^ + *τ* ^2^).

We will use *σ* as our primary measure of model performance (estimated by REML according to the procedure described in the previous section). As the *Q‐*statistic is calculated using weights that does not take into account the between‐study variance, we do not think it is a suitable measure for our purposes. The *I* ^2^ statistic measures the variation across effect sizes that is due to systematic heterogeneity rather than sampling variance (in percent; Thompson & Higgins, 2002). However, while it contains interesting information, *I* ^2^ is not an absolute measure of heterogeneity (Borenstein et al., 2017), and therefore less well suited than *σ* to guide our comparison. The *ω* ^2^ and *τ* ^2^ may be sensitive to the choice of *ρ* in the CHE model, while *σ* is typically not sensitive to this choice (Pustejovsky & Tipton, 2022; Williams et al., 2022). Lastly, as *σ* measures total heterogeneity, we believe it is a better choice of comparison metric than *ω* ^2^ and *τ* ^2^, or *ω* and *τ* (which are otherwise also on the same scale as the effect size estimates). For presentation purposes, we will also report the percentage reduction in estimated effect variance (Williams 2022).

The *ω* ^2^ and *τ* ^2^ statistics need to be estimated, and can be estimated by different algorithms (Veroniki 2016). As mentioned, we will use the REML procedure in the *metafor* package to estimate *ω* ^2^ and *τ* ^2^ (and report *ω* and *τ*).

We will use two analytic approaches that compare model performance. In the first approach, we intend to use a three‐step procedure:

1. We fit the ML method to our data.
2. We select, at most, the nine moderators deemed most important by the ML method in step 1.
3. We include the nine moderators selected in step 2 in a regular meta‐regression and estimate the heterogeneity parameters.

Although the number of moderators to include in a meta‐regression sometimes can be larger than the number of studies divided by 10, this Cochrane Handbook rule of thumb is well‐known and an often used cutoff. However, if the included meta‐analyses indicate that more moderators can be reliably included in meta‐regressions, we may increase the number of moderators. If the ML method indicates that fewer than nine moderators are important, we will include the number deemed to be important. The moderators may include theoretically motivated interactions, in which case we will also include the base variables for the interactions in the meta‐regression (as the association between effect sizes and the base variables is unlikely to be exactly zero). Some ML methods (e.g., random forests) may find interactions ‘automatically’, without the analyst pre‐specifying them, whereas methods like LASSO only performs variable selection among the variables included in the model. We will prespecify all theoretically motivated interactions that we can extract information about from the included studies and include them in the model.

This type of comparison will have the advantage of yielding results that can be readily interpreted and easy to use for other meta‐analysts or intervention researchers when considering, for example, the power of their studies. It will also allow us to report and compare methods across all heterogeneity statistics, which convey different aspects of heterogeneity. Lastly, some ML methods, like metaCART (Li et al., 2017, 2019, 2020), have been applied in this way. MetaCART can be used as a combination of decision trees and sub‐group/moderator meta‐analysis, where the terminal nodes of the tree are treated as subgroups. When the decision tree has been fit, effect sizes are placed into subgroups, according to which terminal node they ended up in and these subgroups are then used for standard subgroup meta‐analysis or, in our case, to develop moderators to include in a metaregression.

Although this procedure seems like a good combination between the performance of ‘black‐box’ ML methods and the interpretability of meta‐regression, there are disadvantages. Doing inference on a subset of moderators that were selected using some statistical procedure is not straightforward (e.g., Berk et al., 2013; Kuchibhotla et al., 2020; Kuchibhotla et al., 2022; Lee et al., 2016). If possible, we will implement a suitable adjustment procedure (e.g., using the procedure developed for linear regression by Kuchibhotla et al., 2020), but our primary purpose in this comparison is to estimate the heterogeneity parameters, not determining the statistical significance of the moderators. An important disadvantage with using the linear‐in‐parameters functional form implied by the meta‐regression framework is that more complex non‐linearities may be obscured. Furthermore, ML methods may indicate that more than nine moderators are important, which would be an interesting result in itself.

Therefore, we will also use a second approach, applied in Williams et al., 2022. They use MetaForest to predict each effect size, then enter this predicted effect size in a meta‐regression as the only moderator. As the predicted effect sizes capture any underlying nonlinearity and still allows estimation of the regular heterogeneity parameters, this approach avoids some of the disadvantages with using the linear‐in‐parameters functional form. The downside with the predicted effect sizes‐approach is that we do not get easily interpretable coefficient estimates for the individual moderators. Furthermore, by comparing the results between the linear‐in‐parameters meta‐regression and the predicted effect sizes meta‐regression, we may learn more about the relation between effect sizes and moderators (e.g., if the results differ, it may be because the functional form is not linear‐inparameters). We, therefore, believe that the two approaches will complement one another. For comparison, we will also conduct the same predicted effect sizes approach using the regular meta‐regression specification.

##### Comparing the predictive performance of ML methods

We will also compare the predictive performance of the ML methods in‐sample and out‐of‐sample. For the in‐sample performance, we will use the data set from Dietrichson et al. (2020, 2021) and follow the recommendations in Raschka (2018): we will use *k*‐fold cross‐validation to find the best model of each ML method, fit this model on the full sample, and calculate the predictive accuracy. Fitting the final model on the full sample—that is, using repeated *k*‐fold cross‐validation without an independent test set, uses as much information as possible, which may be advantageous when the data set is not large (Raschka 2018). As we have a small data set compared to many machine learning applications, and plan to test the performance out‐of‐sample, in a completely independent test data set, we believe this procedure will make best use of our data.

We will also fit the best performing model of each ML method on a new sample of tutoring interventions that were not used to train the ML methods. We will code at most ten new interventions that were published after the search period in Dietrichson et al. (2020, 2021), use the coded information to construct moderators, and then use the results from the meta‐regression and ML methods, estimated on the already existing sample of tutoring interventions, to predict the individual effect sizes in the new studies, as well as the weighted average effect size. The performance metric for these two comparisons will be the root mean square error (RMSE) or, if all methods allow for such an implementation, the weighted RMSE:

(1) $$wRMSE=\sqrt{\left(\sum_{k}w_{k}\left[g_{k}-f{\left(x_{k}\right)}^{2}\right]\right)},$$

where *w*_*k*_ is either a weight supplied by the ML method, or estimated by the CHE model (depending on whether weights need to be estimated outside the ML method in question, see below for more discussion), *g_k_* is effect size, and *f*(*x_k_*) is a function of moderators.

##### Implementation of ML methods

ML methods typically involve training the algorithm on one part of the sample (the training sample) and then using the trained algorithm to predict values of a hold‐out, test, or validation sample (Raschka, 2018). We will use cross‐validation for training and to select the best version of the ML algorithm, while controlling for overfitting.

Cross‐validation is a technique that aims to estimate how well the predictions of a given model will generalize to new samples (Arlot & Celisse, 2010). To implement cross‐validation, we will use a resampling method, which divides data into a pre‐set number of random partitions, *k* = 1, 2, …, *K*, of the data, called folds. For each *k*, the model of choice is then fit on all observations not belonging to *k*. The fitted model is then used to predict the outcome values of all observations in *k*. The model fit measure or measure of error is then computed for the prediction made for *k*. The process is repeated for all folds and the error for each fold is aggregated, resulting in an overall estimate of the out‐of‐sample error or performance for the model of choice. For example, metaCART uses (variants of) the *Q* statistic to decide its splits (Li et al., 2017, 2019, 2020). If possible, we will use the *RMSE* or *wRMSE* as the measure of error or predictive performance. If not possible, we will use the recommended measure for the method in question. As we have a small data set, leave‐one‐out‐cross‐validation (LOOCV) may be computationally possible and advantageous, as it decreases bias (Raschka, 2018). However, it may also increase variance (Raschka, 2018), so we will also try at least one other choice of fold size.

Cross‐validation is thus used in the same way that the Akaike Information Criteria (AIC) and Bayesian Information Criteria (BIC) are usually used, that is, as measures of model fit. While cross‐validation is more computationally demanding, it is also a simple and non‐parametric procedure. The procedure only requires the model in question to be able to predict the outcome value of new observations, given their covariate values. In contrast, information criteria generally requires the number of estimated parameters and the log‐likelihood value of estimated parameters. For a range of machine learning models, such as random forest and K‐nearest neighbours, the number of estimated parameters and the likelihood values are either hard or impossible to compute. Thus, cross‐validation poses as a more ‘model agnostic’ way to control overfitting and can accommodate virtually any method that can produce predictions of an outcome (Arlot & Celisse, 2010).

Some ML methods allow a choice of meta‐analysis model. For example, in the R package *metaforest* (van Lissa, 2020), there is a choice between uniform, fixed, or random‐effects models, and the accompanying weights can be chosen by cross‐validation (i.e., the weights with the best fit are chosen). However, as we believe our data are closer to the assumptions underlying the random‐effects model than a uniform or fixed‐effects model, we will prespecify a random‐effects model for all ML methods (if they allow such a specification).

Many ML methods require a choice of hyperparameters or tuning parameters. Examples include the penalty parameter in LASSO (James et al., 2017), and the number of candidate variables considered at each split of a tree and the minimum number of retained cases in each post‐split group in random forests and MetaForest (van Lissa 2017; van Lissa 2020a). We will use cross‐validation to select the values of hyperparameters.

##### Dealing with missing and interdependent data

Some theoretically interesting moderators have missing values in our data set. To be able to examine such moderators, we will impute missing values. While some ML methods may have built‐in imputation features, not all do and the CHE model does not. To ascertain that all methods use the same data, we will pre‐impute missing values using the *mice* package in R (first developed by van Buuren & Groothuis‐Oudshoorn, 2011), which uses chained equations to predict missing values. We will use the predictive mean matching method to assign missing values. Multiple imputation is generally preferred to single imputation, but we anticipate that running ML methods may be time‐consuming, which may force us to use single imputation. In that case, we will use the average over five data sets to impute a single value.

Most studies in our sample of tutoring interventions reported multiple effect sizes. Either the same students were tested with multiple tests or studies implemented more than one treatment. For both reasons, effect sizes are unlikely to be independent in our sample. Dependence creates problem for statistical inference (e.g., Hedges et al., 2010), but it may also present problems for ML methods. With random forests for example, dependent data may lead to an under‐estimation of the estimated out‐of‐bag error (Janitza et al., 2018). While the CHE models are constructed to account for such dependencies, and the MetaForest method takes this problem into account by using clustered bootstrap sampling (van Lissa, 2020), some ML methods may not have this option (see e.g., van Lissa 2017, for a discussion).

As this potential disadvantage of some ML methods should be taken into account in our comparisons, we would like to keep the structure of our data set intact instead of selecting one effect size from each study, or calculating an average effect size by study. To avoid giving studies with more effect sizes undue weight, which may make it hard for an ML method to compete, we could estimate the between‐study variance using the CHE model, use the estimate to assign each study a weight, divide this study weight between the effect sizes in the study, and then ‘feed’ this within‐study effect size weight to the ML methods that do not take dependencies into account (see Hedges et al., 2010 for a similar suggestion in another setting). When tuning the hyperparameters, we will use cross‐validation strategies that take the hierarchical nature of the data into account (Roberts et al., 2017).

## CONTRIBUTIONS OF AUTHORS

Jens Dietrichson is the lead reviewer on this review. Elizabeth Bengtsen, Trine Filges, Rasmus Klokker, and Terri Pigott are the co‐authors. The responsibilities of the authors are given below:

- Content: Jens Dietrichson, Trine Filges, Rasmus Klokker, and Terri Pigott.
- Systematic review methods: Jens Dietrichson and Trine Filges.
- Statistical analysis: Jens Dietrichson, Trine Filges, Rasmus Klokker, and Terri Pigott.
- Information retrieval: Elizabeth Bengtsen.

## DECLARATIONS OF INTEREST

The tutoring‐data set used in this review is a subset of the studies included in Dietrichson et al. (2020, 2021), where Dietrichson, Klokker, and Filges were among the co‐authors. Dietrichson is a co‐author on one of the studies included in the tutoring data set (Bøg et al., 2021).

## PRELIMINARY TIMEFRAME

The approximate date for submission of the systematic review is December 2025.

### PLANS FOR UPDATING THIS REVIEW

Jens Dietrichson will be responsible for updating this review as funding becomes available.

## SOURCES OF SUPPORT

### Internal sources

- VIVE Campbell, Denmark

### External sources

- No external support. Other.

## Supporting information

Supporting information.
