# Supplementary material for: Protocol: Machine learning for selecting moderators in meta‐analysis: A systematic review of methods and their applications, and an evaluation using data on tutoring interventions
Source: Campbell Syst Rev. 2024 Dec 10;20(4):e70009. doi: 10.1002/cl2.70009 (PMC11632158; doi:10.1002/cl2.70009)
Supplement: Supplementary file 1 — Supporting information. [file CL2-20-e70009-s001.docx]

**Appendices**

**Appendix 1. Appendix A: Coding scheme for tutoring studies**

We plan to extract the following information from all tutoring studies that we will include in the meta-analysis.

1. **Report characteristics**
   1. Authors
   2. Title
   3. Publication year
   4. Language
   5. Publishing status (published in scientific journal or not)
   6. Outlet/Type of publication (e.g., journal name/report series/dissertation)
2. **Study characteristics**
   1. Study location (country and state/city)
   2. Study design (RCT, QRCT, QES)
   3. Describe treatment assignment
   4. Number of separate sites included in the study (classrooms, schools, districts, states orregions, countries)
   5. If multiple sites, describe if there were differences in assignment between sites?
3. **Participant characteristics**
   1. Describe the target group of the intervention, e.g., students with specific learning difficulties, lowachievement, low SES etc.
   2. Gender (share of girls)
   3. Age distribution (min, max, mean)
   4. Grade distribution (min, max, mean)
   5. Ethnic, cultural, and language background (share of students with a differentbackground than the majority student population)
   6. Socioeconomic status (share low income, share low parental education, share low status parentaloccupation)
   7. Did the sample include the students with most difficulties or were there some form of cutoff used (e.g.,students' scoring below a certain threshold/with a certain type of difficulty were not included)?
4. **Intervention characteristics**
   1. Name of intervention
   2. Describe the instructional methods used in the intervention and any differences between intervention andcontrol groups regarding these methods. State explicitly if there are no differences.
   3. Describe the content domain targeted by the intervention and any differences between treatment and controlgroups regarding the content they are instructed in. State explicitly if there are no differences.
   4. Intervention site (school/other, in which case, specify)
   5. Group size (e.g. 1:1, 1:2, …)
   6. Intervention implementer (Describe the person(s) delivering the intervention: e.g., teachers, college students,researchers).
   7. Is the implementer trained, and for how long? (the number of hours/days/weeks).
   8. Duration of intervention in weeks (intended, received)
   9. Frequency of intervention in sessions (intended, received)
   10. Intended intensity of intervention in hours per week (intended, received)
   11. Implementation quality (questions from Wilson, Lipsey, Tanner-Smith, Huang, & Steinka-Fry, 2010): Wasthe implementation of the programme monitored by the author/researcher or programme personnel to assess whether it was delivered as intended? (Yes/No/Cannot tell)
   12. Based on evidence or author acknowledgement, was there any uncontrolled variation or degradation inimplementation or delivery of treatment, e.g., high dropouts, erratic attendance, treatment not delivered as intended, wide differences between settings or individual providers, etc.? Assume that there is no problem if one is not specified (yes (describe below)/ possible (describe below)/ no, apparently implemented as intended)
   13. Describe implementation problems, if any.
   14. Describe the degree of structure/how manual based the intervention is. E.g., were tutors asked to follow amanual, or were there scripted lessons/sessions.
   15. Did the intervention employ any specific methods, such as multisensory instruction or instruction instudents' mother tongue?
5. **Control/Comparison characteristics**
   1. What is the nature of the control/comparison condition? (Controls do not receive anyintervention/treatment/service after the end of intervention; Controls are wait-list controls)
   2. If the control group receives a treatment as usual condition, did this condition include any supplementaryinstruction/services, or just regular classroom instruction?
6. **Outcome measurement (each step is repeated for all relevant outcomes)**
   1. Measurement timing (number of months after end of intervention)
   2. Name of standardised test
   3. Subject of standardised test (mathematics, reading)
   4. Content domain(s) of test (e.g., vocabulary, algebra, general etc)
   5. Who performs the tests?
7. **Sample size**
   1. Sample size used in analysis for outcome measurement (separate for intervention and control groups)
8. **Outcomes**
   1. Test score (Code for both pre- and post-intervention tests separate for intervention and control groups ifpossible. Describe test in terms: continuous/dichotomous; whether high score/1 is desirable; type of statistic, e.g., mean, t-test, beta-coefficient, F-test etc)
   2. Standard deviation (Code for both pre- and post-intervention tests separate for intervention and controlgroups if possible)
   3. Estimation method (e.g., raw means, adjusted means, regression adjusted etc)
   4. Uncommon standardisation (No/Mention method of standardisation if available standard deviation is otherthan the raw standard deviation for the intervention and control group)
